# Agreement between cardiovascular disease risk assessment tools: An application to the United Arab Emirates population

**DOI:** 10.1371/journal.pone.0228031

**Published:** 2020-01-24

**Authors:** Abderrahim Oulhaj, Sherif Bakir, Faisal Aziz, Abubaker Suliman, Wael Almahmeed, Harald Sourij, Abdulla Shehab

**Affiliations:** 1 Institute of Public Health, College of Medicine & Health Sciences, United Arab Emirates University, Al Ain, United Arab Emirates; 2 Zayed Center for Health Sciences, United Arab Emirates University, United Arab Emirates; 3 Cardiology Department, Sheikh Shakhbout Medical City, United Arab Emirates; 4 Cardiovascular Diabetology Research Group, Division of Endocrinology and Diabetology, Department of Internal Medicine, Medical University of Graz, Graz, Austria; 5 Center for Biomarker Research in Medicine (CBmed), Graz, Austria; 6 Heart and Vascular Institute, Cleveland Clinic, Abu Dhabi, United Arab Emirates; 7 Department of Internal Medicine, College of Medicine & Health Sciences, United Arab Emirates University, Al Ain, United Arab Emirates; Karolinska Institutet, SWEDEN

## Abstract

**Introduction:**

Evidence regarding the performance of cardiovascular disease (CVD) risk assessment tools is limited in the United Arab Emirates (UAE). Therefore, we assessed the agreement between various externally validated CVD risk assessment tools in the UAE.

**Methods:**

A secondary analysis of the Abu Dhabi Screening Program for Cardiovascular Risk Markers (AD-SALAMA) data, a large population-based cross-sectional survey conducted in Abu Dhabi, UAE during the period 2009 until 2015, was performed in July 2019. The analysis included 2,621 participants without type 2 Diabetes and without history of cardiovascular diseases. The CVD risk assessment tools included in the analysis were the World Health Organization for Middle East and North Africa Region (WHO-MENA), the systematic coronary risk evaluation for high risk countries (SCORE-H), the pooled cohort risk equations for white (PCRE-W) and African Americans (PCRE-AA), the national cholesterol education program Framingham risk score (FRAM-ATP), and the laboratory Framingham risk score (FRAM-LAB).

**Results:**

The overall concordance coefficient was 0.50. The agreement between SCORE-H and PCRE-W, PCRE-AA, FRAM-LAB, FRAM-ATP and WHO-MENA, were 0.47, 0.39, 0.0.25, 0.42 and 0.18, respectively. PCRE-AA classified the highest proportion of participants into high-risk category of CVD (16.4%), followed by PCRE-W (13.6%), FRAM-LAB (6.9%), SCORE-H (4.5%), FRAM-ATP (2.7%), and WHO-MENA (0.4%).

**Conclusions:**

We found a poor agreement between various externally validated CVD risk assessment tools when applied to a large data collected in the UAE. This poses a challenge to choose any of these tools for clinical decision-making regarding the primary prevention of CVD in the country.

## Introduction

Cardiovascular disease (CVD) is a leading cause of morbidity and mortality, contributing to 203 million disability-adjusted life years and 31% of deaths worldwide [1]. The number of CVD-related deaths is expected to increase from 17.7 million in 2015 to 23.6 million in 2030 [1]. In the United Arab Emirates (UAE), CVD contributes to 40% of all deaths and its incidence is increasing in young adults mainly because of an increase in the burden of its known risk factors such as abdominal obesity (71.5%), dyslipidemia (74.0%), hypertension (43.0%), and diabetes (32.4%) [2,3].

Primary prevention of CVD is considered as the most beneficial and cost-effective intervention strategy because of the high burden and treatment cost of CVD. Therefore, international clinical guidelines recommend incorporating CVD risk assessment tools into routine clinical practice for accurate identification and subsequent evidence-based treatment of individuals at a high risk of developing CVD [4].

At present, various externally validated CVD risk assessment tools are being used for primary and secondary prevention of CVD in the UAE. The list of these tools includes, but is not limited to, the systematic coronary risk evaluation (SCORE) charts used by the European Society of Cardiology and the European Atherosclerosis Society guidelines [5], the Pooled Cohort risk equation (PCRE) used by the American College of Cardiology/American Heart Association guidelines [6], the Framingham risk score for hard CHD used by the National Cholesterol Education Program-Adult Treatment Panel-III (FRAM-ATP) [7], the laboratory Framingham risk score for CVD (FRAM-LAB) [8], and the World Health Organization risk charts for Eastern Mediterranean and North Africa Region [9]. Except the WHO-MENA CVD risk chart, no other tool has yet been developed, validated, or calibrated in the UAE [10,11]. In addition, there is insufficient data on the performance and agreement between different externally validated CVD risk assessment tools that are currently used in the UAE. To the best of our knowledge, no study has been yet conducted in the UAE to explore this issue. Therefore, we conducted this analysis to assess the agreement between a variety of externally validated CVD risk assessment tools in the UAE. We also classified subjects into the high-risk category according to the thresholds of each risk assessment tool and then compared the percentages of high-risk subjects between different risk assessment tools.

## Methods

### Study design

We performed a secondary analysis of the Abu Dhabi Screening Program for Cardiovascular Risk Markers (AD-SALAMA) data. The AD-SALAMA is a large population-based cross-sectional survey, designed and conducted by the Cardiac Sciences Institute of Sheikh Khalifa Medical City (SKMC) in Abu Dhabi, UAE. The primary objective of this survey was to estimate the burden of major risk factors of CVD, to identify individuals at high risk for developing CVD, and to improve primary prevention of CVD. The study population included adult (25–75 years) men and women of different ethnic backgrounds who were working at various governmental and non-governmental entities in Abu Dhabi. Subjects were randomly invited to attend an educational event about cardiovascular disease risk factors where they were asked to voluntarily participate in the study.

### Data collection and measurements

After obtaining the permission from participating entities, trained nurses of SKMC screened volunteer employees at either mobile clinics or visiting each entity on scheduled days. A total of 6069 subjects were screened during the period 2009 until 2015 at different governmental and non-governmental entities. Demographic and clinical data included age (years), sex, smoking, diabetes, height, weight, body mass index (BMI), systolic and diastolic blood pressure, hypertension, and use of hypertensive drugs. Blood samples were also collected to measure total cholesterol (TC), high-density lipoprotein cholesterol (HDL-C), low-density lipoprotein cholesterol (LDL-C), triglyceride (TG), and glucose levels.

### Ethical considerations

The study adhered to the ethical principles of the Declaration of Helsinki and was approved by Sheikh Khalifa Medical City (SKMC) human ethics research committee. Both verbal and written Informed consents were obtained from the study participants before data collection.

### Data extraction

We computed the agreement in a sub-population of AD-SALAMA subjects without diabetes, without established CVD and those aged between 40 and 70 years. The reason for excluding diabetic subjects from the analysis is because they are already considered as high-risk subjects by most guidelines and also in order to make a fair comparison between different risk assessment tools as some of them do not use type 2 Diabetes as a predictor. The subjects with established CVD were excluded because we are investigating primary not secondary prevention. Therefore, we excluded 521 with type 2 diabetes mellitus, 1708 aged less than 30 or more than 70 years, and 1219 subjects with missing values in predictor’s variables. This led to 2621 subjects with complete data on risk factors required for computing CVD risks. None of the subjects considered for the final analysis were under lipid lowering therapy.

### CVD risk assessment tools

The CVD risk assessment tools included in the analysis were the WHO risk chart for the Eastern Mediterranean and North Africa region (WHO-MENA) [2], SCORE for high-risk countries (SCORE-H) [5], PCRE for White (PCRE-W), PCRE-AA (PCRE for African American) [6], FRAM-ATP (used in NCEP-ATP-III) [7], and the laboratory Framingham risk score for CVD (FRAM-LAB) [8], Details on the geographical location, age range, risk factors used for the derivation of these risk assessment tools, the corresponding guidelines, and the types of outcomes are reported in Table 1.

**Table 1:** Characteristics of cardiovascular risk assessment tools.

Risk tools	Guidelines	Location/Cohort	Age	Parameters	Outcomes
WHO-MENA	WHO	Middle Eastern Countries	40–70 years	Age, gender, smoking, SBP, TC, DM	10-year risk of fatal or non-fatal MI or stroke
SCORE-H	ESC/EAS	12 European cohorts	40–65 years	Age, gender, SBP, TC, HDL-C, smoking, region	10-year risk of CHD death or stroke death
PCRE-W PCRE-AA	ACC/AHA	CARDIA, Framingham, ARIC, CHS, USA	40–79 years	Age, gender, SBP, HTN treatment, TC, HDL-C, DM, smoking	10-year risk of fatal or non-fatal CHD or fatal or non-fatal stroke
FRAM-ATP III	NCEP-ATPIII	Framingham Heart Study	20–79 years	Age, gender, TC, smoking, HDL-C, SBP, HTN treatment	10-year risk for myocardial infarction and coronary death
FRAM-LAB		Framingham, MA, USA	30–74 years	Age, gender, SBP, HTN treatment, TC, HDL-C, DM, smoking	10-year risk of coronary death, MI, coronary insufficiency, angina, ischemic stroke, hemorrhagic stroke, transient ischemic attack, peripheral artery disease, or heart failure

ACC: American College of Cardiology, AHA: American Heart Association, ARIC: The Atherosclerosis Risk in Communities, ATP III: Adult Treatment Panel III, CARDIA: Coronary Artery Risk Developing in Young Adults, CHS: Cardiovascular Health Study, DM: Diabetes Mellitus, FRAM: Framingham, HDL-C: High-Density Lipids-Cholesterol, HTN: Hypertension, MENA: Eastern Mediterranean and North Africa Region, MI: Myocardial Infarction, NCEP: National Cholesterol Education Program, PCRE: Pooled Cohort Risk Equations, SBP: Systolic Blood Pressure, SCORE: Systematic Coronary Risk Evaluation, TC: Total Cholesterol, WHO: World Health Organization.

### Statistical analysis

Descriptive statistics for quantitative and qualitative variables were provided to describe the characteristics of the population under study. Predicted 10-year CVD risks were computed using the original equations provided in their corresponding published papers. CVD risk equations derived from original publications were calculated using an online application developed by the corresponding author. The online application is based on the R Package Shiny and can be freely accessed through the following link [https://comparecvdandguidelines.shinyapps.io/internal-private-test/]. Overall and pairwise Lin’s concordance correlation coefficients (CCC) were calculated to measure the degree of agreement overall and between each pair of risk assessment tools [12,13]. Lin’s concordance coefficient quantifies the level of agreement between risk tools and ranges between -1 and 1 with the upper limit considered as a perfect agreement. The rule of thumb for a good level of agreement is a value of CCC over 0.9 [14]. We also calculated the percentage of subjects assigned to the high-risk category according to the threshold provided in the corresponding original guideline for each risk assessment tool. The thresholds used were 20% for WHO-MENA, 5% for SCORE-H, 7.5% for PCRE-W and PCRE-AA, 20% for FRAM-ATP, and 20% for FRAM-LAB [5–8,10]. In this analysis, we also assessed discrepancies between different risk assessment tools by reporting misclassification rates. This was investigated by calculating the percentage of low-risk subjects who were re-classified as high-risk according to other tools. In order to do this, the European risk assessment tool SCORE-H was used as a reference and was compared against all other risk assessment tools. The current analysis involves individuals with complete data on all risk factors required for the calculation of different risk scores. As sensitivity analysis, we used multiple imputation to make sure that missing data excluded from the current analysis does not affect the validity of the results. We re-run the whole analysis by imputing missing data using the “Multivariate Imputation by Chained Equations” method implemented in the mice package in R. All statistical analysis and data manipulation were performed in December 2019 using the R software version 3.5.2.

## Results

### Characteristics of participants

Of the total number of subjects (N = 2,621), 558 (21%) were females and 221 (8.4%) were hypertensive. The demographic and clinical characteristics of this population are summarized overall and by gender in Table 2.

**Table 2:** Demographic and clinical characteristics of study participants (N = 2,621).

Variables	Total (n = 2,621)	Female (n = 558)	Male (n = 2,063)
Age—years, Mean (±SD)	40.2 (±8.4)	38.5 (±7.8)	40.7 (±8.5)
Weight–kg, Mean (±SD)	78.7 (±17.0)	69.6 (±16.7)	81.2 (±16.2)
BMI—kg/m^2^, Mean (±SD)	27.3 (±5.4)	26.9 (±7.1)	27.4 (±4.8)
Cholesterol—mg/dl, Mean (±SD)	179.1 (±42.2)	180.6 (±38.0)	178.7 (±43.3)
DBP–mmHg, Mean (±SD)	84.3 (±11.2)	79.4 (±10.9)	85.6 (±10.9)
Glucose—mg/dl, Mean (±SD)	89.8 (±46.4)	86.3 (±44.9)	90.8 (±46.7)
HDL—mg/dl, Mean (±SD)	39.9 (±17.0)	52.9 (±23.4)	36.3 (±12.8)
Height–cm, Mean (±SD)	168.2 (±15.5)	157.5 (±19.1)	171.0 (±13.0)
Hypertension, n (%)			
No	2,400 (91.6%)	531 (95.2%)	1,869 (90.6%)
Yes	221 (8.4%)	27 (4.8%)	194 (9.4%)
LDL—mg/dl, Mean (±SD)	98.7 (±47.3)	92.9 (±42.0)	100.3 (±48.5)
National, n (%)			
No	2,150 (85.0%)	386 (72.4%)	1,764 (88.3%)
Yes	380 (15.0%)	147 (27.6%)	233 (11.7%)
SBP–mmHg, Mean (±SD)	131.6 (±17.7)	121.0 (±16.6)	134.5 (±16.8)
Smoking, n (%)			
No	2,007 (76.6%)	497 (89.1%)	1,510 (73.2%)
Yes	614 (23.4%)	61 (10.9%)	553 (26.8%)
Triglyceride—mg/dl, Mean (±SD)	173.3 (±102.5)	145.7 (±86.5)	180.9 (±105.2)

Continuous variables are summarized as Mean (±SD). Categorical variables are summarized as n (%). BMI: Body Mass Index, DBP: Diastolic Blood Pressure, HDL: High-Density Lipoprotein, LDL: Low-Density Lipoprotein, n: Frequency, SBP: Systolic Blood Pressure, SD: Standard Deviation

Summaries of the 10-year risks of experiencing a CVD event according to each risk assessment tool discussed earlier are presented in Table 3. Except the WHO-MENA, all other risk assessment tools assigned significantly (*p* < 0.001) higher 10-year CVD risk scores to men compared to women. This difference in the average risk between men and women was expected because the CVD risk assessment tools used in this study assigned, in their original equations, a higher risk for men compared to women.

**Table 3:** Estimated 10-year risk of cardiovascular disease according to different risk assessment tools.

Risk Tools	Total (n = 2,621)	Female (n = 558)	Male (n = 2,063)	P-Value
	Mean (±SD)*	Mean (±SD)*	Mean (±SD)*	
NCEP-ATP III	3.6 (±5.0)	0.9 (±1.3)	4.4 (±5.4)	<0.001
Fram-LAB	7.6 (±7.6)	2.7 (±2.7)	8.9 (±7.9)	<0.001
PCRE-AA	4.6 (±4.6)	1.6 (±4.3)	5.4 (±4.3)	<0.001
PCRE-W	3.5 (±4.6)	1.2 (±2.2)	4.1 (±4.8)	<0.001
SCORE-H	1.1 (±2.0)	0.2 (±0.5)	1.3 (±2.2)	<0.001
WHO-MENA	5.3 (±2.2)	5.1 (±1.1)	5.3 (±2.4)	0.018

FRAM-LAB: Framingham-Laboratory, NCEP-ATP III: National Cholesterol Education Program-Adult Treatment Panel III, PCRE-AA: Pooled Cohort Risk Equations-African Americans, PCRE-W: Pooled Cohort Risk Equations-White, SCORE-H: Systematic Coronary Risk Evaluation-High risk countries, SD: Standard Deviation, WHO-MENA: World Health Organization-Eastern Mediterranean and North Africa Region

*Estimated 10-year risk of cardiovascular disease is expressed in percentage (%).

### Agreement between risk assessment tools

**Fig 1:**
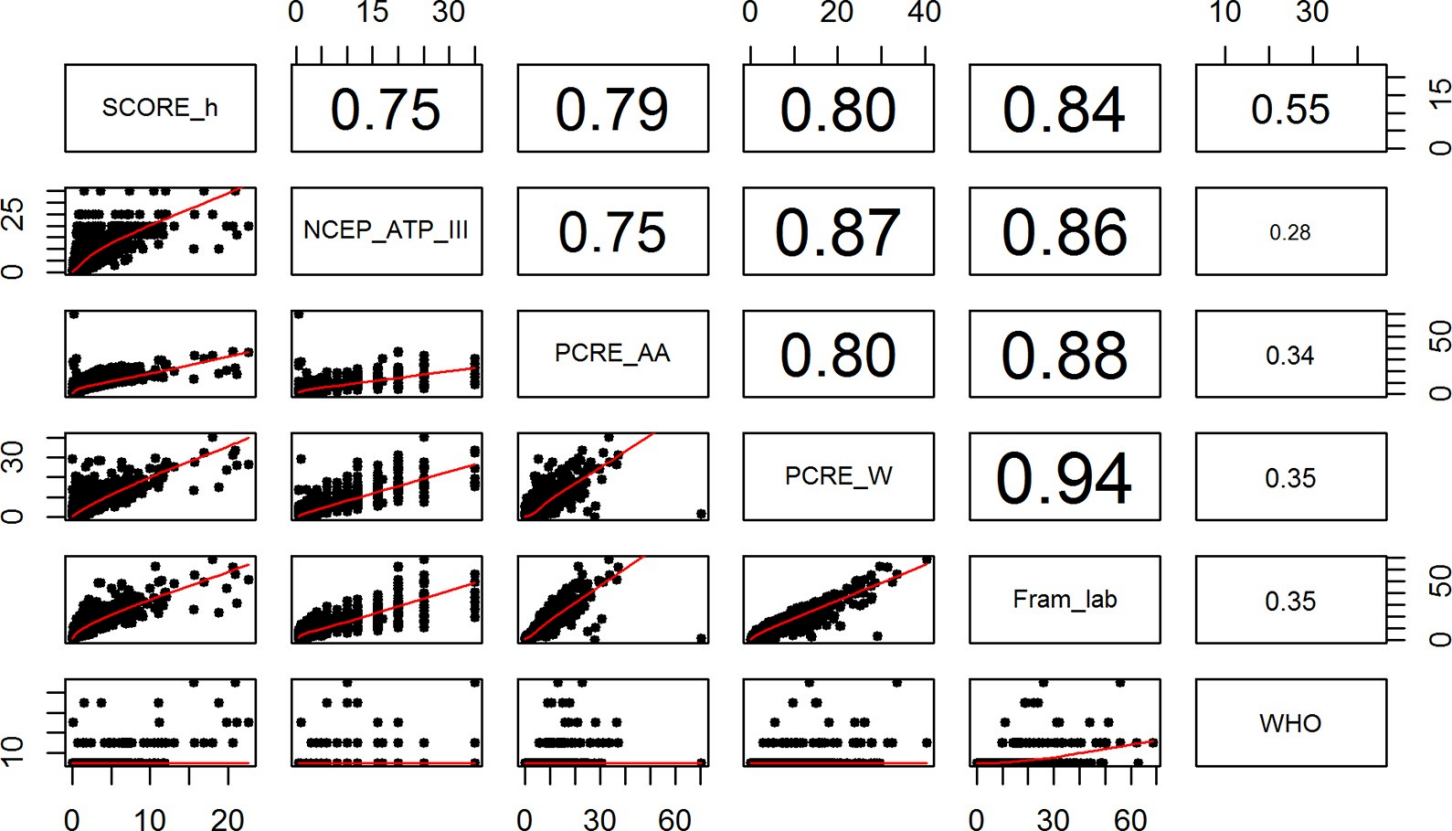
Relationship between different CVD risk assessment tools. FRAM-LAB: Framingham-Laboratory, NCEP_ATP_III: National Cholesterol Education Program-Adult Treatment Panel III, PCRE-AA: Pooled Cohort Risk Equations-African Americans, PCRE-W: Pooled Cohort Risk Equations-White, SCORE-h: Systematic Coronary Risk Evaluation for high risk countries, WHO: World Health Organization.

Despite high values of Pearson’s correlation coefficients between different risk assessment tools, except the WHO-MENA (Fig 1), we found low-to-moderate overall agreement between different risk assessment tools with an overall CCC equal to 0.50. This illustrates the structural difference between the correlation and agreement concepts. In fact, agreement is used for variables that are meant to measure the same construct, while correlation is used for variables that measure completely different constructs. When compared with SCORE-H, the CCCs for FRAM-ATP, PCRE-AA, PCRE-W, FRAM-LAB, and WHO-MENA, were 0.42, 0.39, 0.47, 0.25 and 0.18, respectively. More information on all pairwise Lin’s concordance coefficients is available in Table 4.

**Table 4:** Agreement between all pairs of CVD risk assessment tools.

Risk Tools	SCORE-H	FRAM-ATP-III	PCRE-AA	PCRE-W	FRAM-LAB	WHO-MENA
**SCORE-H**	1	0.42	0.39	0.47	0.25	0.18
**NCEP-ATPIII**		1	0.73	0.87	0.67	0.19
**PCRE-AA**			1	0.78	0.69	0.26
**PCRE-W**				1	0.69	0.25
**FRAM-LAB**					1	0.17
**WHO-MENA**						1

FRAM-LAB: Framingham-Laboratory, NCEP-ATP III: National Cholesterol Education Program-Adult Treatment Panel III, PCRE-AA: Pooled Cohort Risk Equations-African Americans, PCRE-W: Pooled Cohort Risk Equations-White, SCORE-H: Systematic Coronary Risk Evaluation-High risk countries, SD: Standard Deviation, WHO-MENA: World Health Organization-Eastern Mediterranean and North Africa Region

### Identification of high CVD risk subjects according to different risk assessment tools

**Fig 2:**
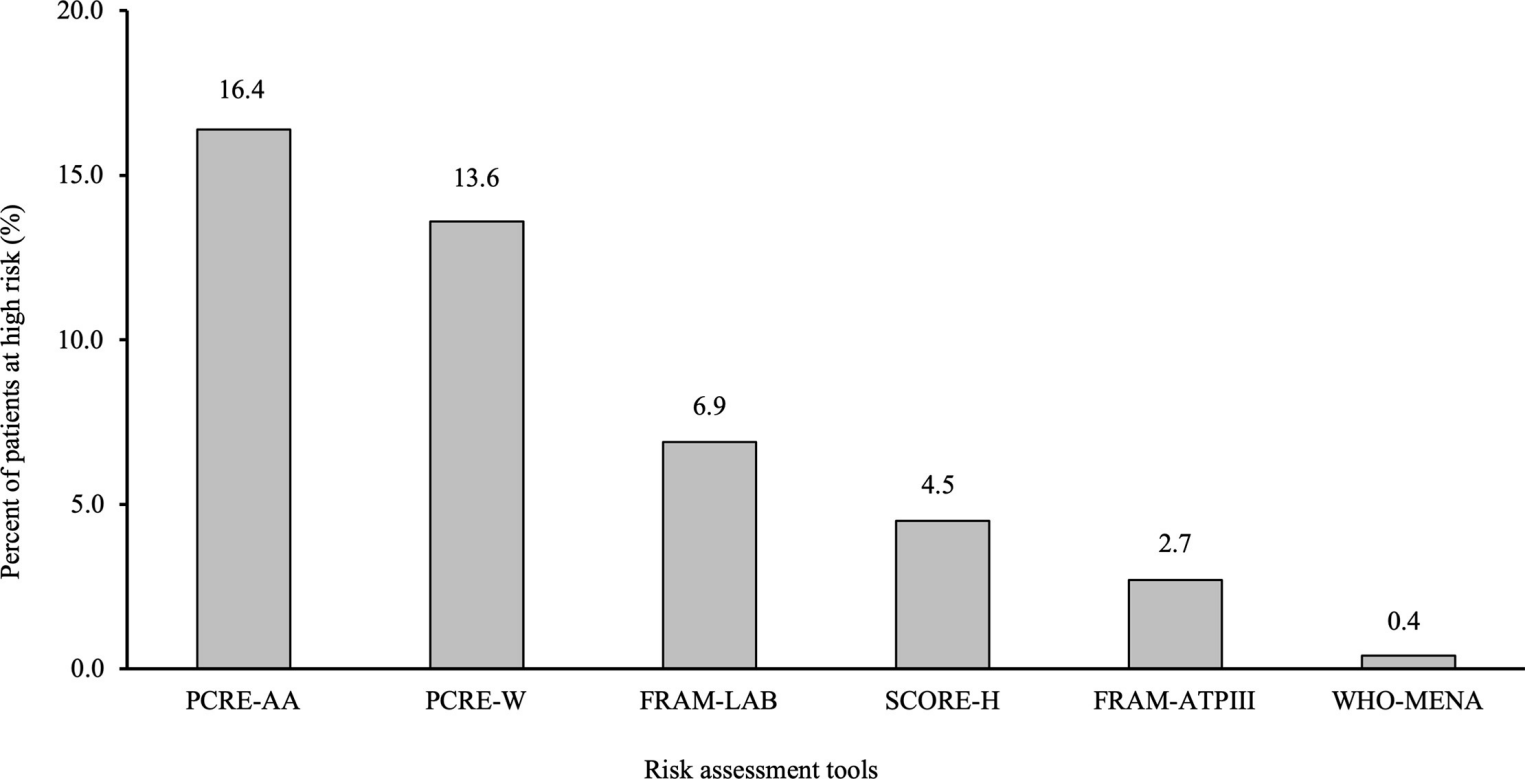
Proportion of subjects classified as high risk (n = 2,621). FRAM-LAB; Framingham-Laboratory, NCEP-ATPIII: National Cholesterol Education Program-Adult Treatment Panel III, PCRE-AA: Pooled Cohort Risk Equations-African Americans, PCRE-W: Pooled Cohort Risk Equations-White, SCORE: Systematic Coronary Risk Evaluation, WHO-MENA: World Health Organization-Eastern Mediterranean and North Africa region.

**Fig 3:**
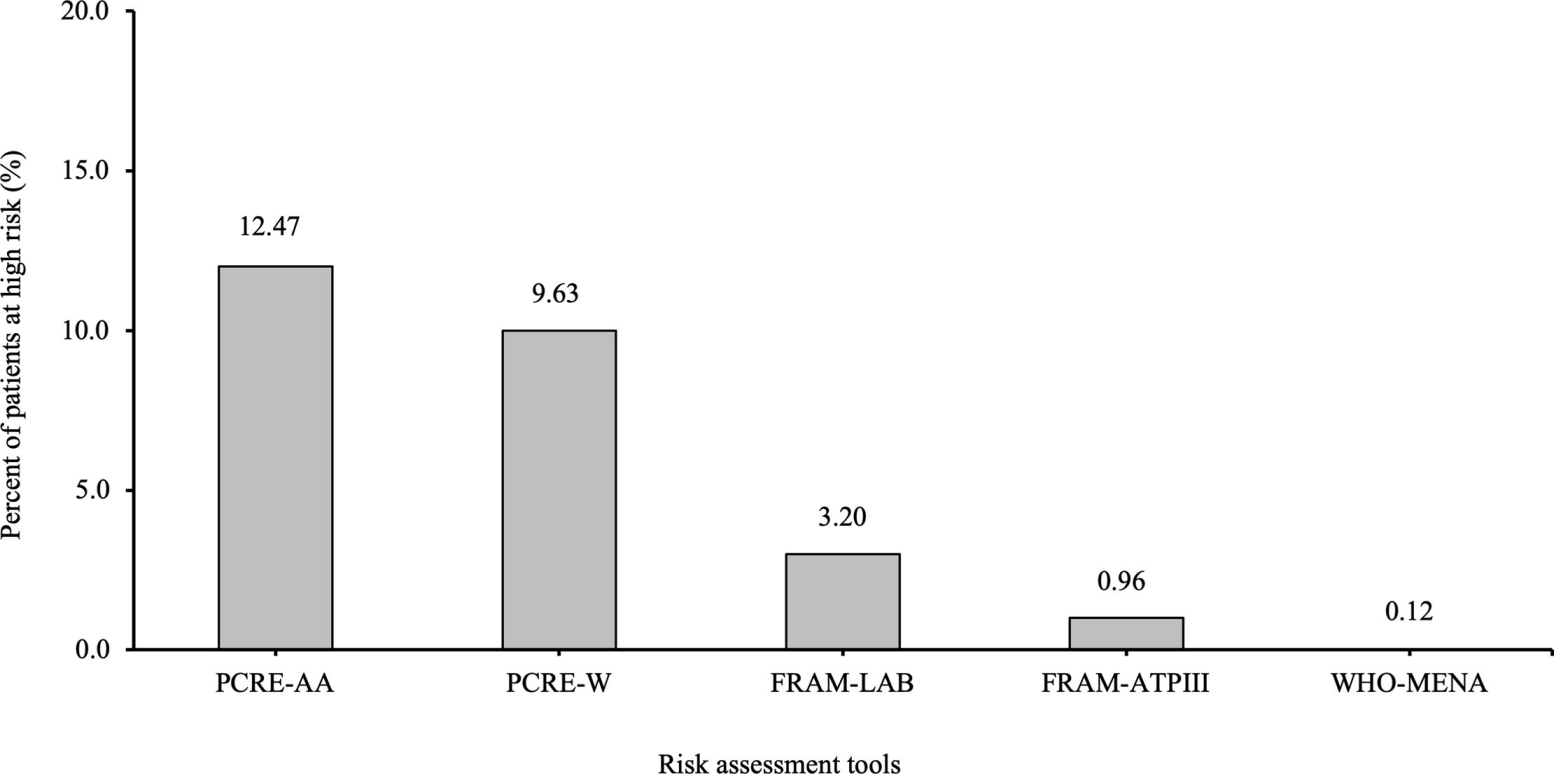
Proportion of subjects classified as low risk according to SCORE-H but reclassified as high risk according to other risk assessment tools (n = 2,502). FRAM-LAB; Framingham-Laboratory, NCEP-ATPIII: National Cholesterol Education Program-Adult Treatment Panel III, PCRE-AA: Pooled Cohort Risk Equations-African Americans, PCRE-W: Pooled Cohort Risk Equations-White, SCORE: Systematic Coronary Risk Evaluation, WHO-MENA: World Health Organization-Eastern Mediterranean and North Africa region.

When each of the risk tools was used with thresholds specified in the guidelines, PCRE-AA classified the highest proportion of participants under the high-risk category (16.4%, 95% confidence interval [CI]: 15.1–17.9%), meaning that 16.4% of these subjects should be assigned to statin therapy. The proportion of subjects classified under the high-risk category according to the remaining tools was 13.6% (95% CI: 12.3–15.0%) for PCRE-W, 6.9% (95% CI: 6.0–8.0%) for FRAM-LAB, 4.5% (95% CI: 3.8–5.4%) for SCORE-H, 2.7% (95% CI: 2.2–3.5%) for FRAM-ATP, and 0.4% (95% CI: 0.2–0.8%) for the WHO-MENA (Fig 2). Furthermore, among the 2502 participants who were classified as low-risk subjects according to SCORE-H, 12.47% (n = 312), 9.63% (n = 241), 3.20% (n = 80), 0.96% (n = 24), and 0.12% (n = 3) were re-classified as high-risk subjects according to PCRE-AA, PCRE-W, FRAM-LAB, FRAM-ATP, and WHO-MENA, respectively (Fig 3).

### Sensitivity analysis

To check whether the exclusion of patients with diabetes from the current analysis led to any bias, we analyzed the data by including these patients. The average risk estimates increased for all risk tools due to the inclusion of diabetic patients. However, the overall agreement (0.49) between risk tools remained similar to the one estimated in patients without diabetes (0.50).

We also re-run the whole analysis by imputing missing data using the “Multivariate Imputation by Chained Equations” method implemented in the mice package in R. The number of imputed datasets was set to 5 and the predictive mean matching was selected as the imputation method. The estimated overall agreement (CCC) in the 5 imputed data sets were very close to each other (0.5033, 0.4934, 0.4953, 0.4950, and 0.4787). Furthermore, all other quantities estimated in the complete case analysis (current analysis) were also very similar in the 5 data sets.

## Discussion

In this study, we assessed the agreement between six CVD risk assessment tools that are commonly used in the UAE population. These risk assessment tools were compared by analyzing the data generated from a large screening program in Abu Dhabi, UAE. The analysis showed a low agreement between risk assessment tools in estimating the 10-year risk of CVD. The probability of classifying subjects into high-risk category of CVD varied substantially between risk assessment tools.

As mentioned earlier, we found low overall agreement (50%) and low-to-moderate pairwise agreement (from 17% to 78%) between various tools. To the best of our knowledge, this is the first study comparing the agreement between CVD risk assessment tools in the UAE. Nonetheless, studies conducted in different populations have also yielded similar results regarding the agreement between CVD risk assessment tools. A systematic review of 20 studies reported wide variations in the level of agreement (from 22% to 100%) between various calculators [15]. Another study showed 14% to 44% agreement between different CVD risk assessment tools in the Peruvian population [16]^.^ The analysis of hypothetical data from eight countries demonstrated the overall agreement of 64% between 25 CVD risk assessment tools [17]. We also noted that the WHO-MENA and the European-based SCORE-H had the lowest pairwise agreement with the US-based FRAM, PCRE, and ATP-III tools. Similar results were also reported in the Peruvian population and the systematic review [15,16].

In this study, the estimated 10-year risk of CVD varied in average from 1.1% to 7.6% between tools. FRAM-LAB and PCRE estimated a higher risk of CVD, whereas SCORE-H, FRAM-ATP-III, and WHO-MENA estimated a lower risk. Consistent with our findings, the average CVD risk estimation was the highest using FRAM and the lowest using WHO in the Indian population [18]. Conversely, PCRE showed the highest CVD risk, followed by FRAM, and SCORE estimated the lowest risk in Northern Iran [19]. Like CVD risk estimation, the probability of assigning participants to the high-risk category for CVD also varied significantly between risk assessment tools in our study. As such, the proportion of subjects classified into the high-risk category as per PCRE was 6 times higher than those as per FRAM-ATP-III and WHO-MENA. Similar to our results, the highest proportion of individuals were classified to be at high risk of CVD as per PCRE, followed by FRAM (non-lab) and SCORE in the Peruvian and Iranian population [16,19]. According to another study in India, the FRAM tool assigned more individuals to high CVD risk category compared to PCRE [18,20]. Despite the inconsistencies in CVD risk estimation among tools, the WHO tool has consistently produced the worst CVD risk estimation and classification in Malaysian, Indian, Peruvian, and the UAE populations [16,18,21].

A vast number of validation studies has confirmed the inconsistency in CVD risk evaluation between tools. The FRAM tool over-estimated the risk of fatal and non-fatal CVD when used in non-US populations [15,22,23]. Likewise, the PCRE has been reported to over-estimate the risk of CVD in Chinese and under-estimate the risk in South Asian American populations [22,24]^.^ Consequently, these tools assign more individuals to lipid-lowering therapy compared with other tools. In contrast, both SCORE-H and WHO tools tend to under-estimate the risk of CVD in various populations worldwide, making a small proportion of individuals eligible to lipid-lowering therapy [15,23]. These disagreements suggest that clinicians should be wary of estimation differences presented by each tool before using them in clinical practice. In addition, it also means that there is a need of validating and/or recalibrating CVD tools in target populations to enhance the accuracy of these tools.

Many factors may have been responsible for the poor agreement between CVD risk assessment tools in our study. One such factor is the risk classification threshold, which differs among tools. For instance, the threshold to assign subjects to the high-risk category of CVD is 20% for FRAM-LAB, WHO-MENA, and FRAM-ATP, 7.5% for PCRE and 5% for SCORE-H [5–8,10]. The tools also differ from each other with regard to their CVD endpoints. FRAM-LAB and PCRE incorporate the risk for both fatal and non-fatal CVD events in their risk equations. SCORE-H estimates the risk of fatal CVD events only, FRAM-ATP-III predict the occurrence of hard CHD events, and the WHO tool includes both fatal and non-fatal myocardial infarction and/or stroke [5–8,10]. As CHD is a subset of CVD, the tools measuring only CHD have lower estimated risks than those measuring total CVD. This may explain why SCORE-H and ATP-III predicted lower CVD risks than those predicted by FRAM and WHO in our study. But since the agreement is based on the ranks rather than the absolute values of the risks, this argument might be not plausible. Poor agreement between CVD risk assessment tools can also occur because of source cohorts from which the individuals risk assessment tools are generated. The SCORE tool is derived from multiple European cohorts, whereas FRAM, NCEP-ATP-III, and PCRE are US-based tools generated from Framingham Heart Study, CARDIA, ARIC, and CHS cohorts [5–8,10]. These baseline cohorts differ vastly with respect to burden of underlying risk factors and CVD outcomes, therefore, resulting probably in a poor agreement of risk estimation and classification among tools. In our study, this may have been one of many reasons for a low level of agreement between the European SCORE-H tool and the US-based tools and moderate agreement among US-based tools. In comparison, WHO-MENA is based on a hypothetical cohort generated from the research evidence in Eastern Mediterranean countries [2]. Therefore, the WHO tool has shown poor risk estimation, correlation, and agreement with other tools in this study and previous research [15,23]. Type of risk factors included in risk equations are also responsible for the variation in risk prediction across CVD tools. In addition to other factors, FRAM-LAB, FRAM-ATP-III, and PCRE include hypertension (HTN) treatment, HDL cholesterol, and total cholesterol in their equations, whereas WHO does not include HTN treatment and HDL cholesterol and SCORE does not include HTN treatment in its risk equation [5–8,10].

### Limitations

We used a cross-sectional data to compare the agreement between risk assessment tools. Therefore, we could not estimate the actual 10-year risk of CVD in the UAE. The low observed level of disagreement might be due to the heterogeneity in the primary outcomes between risk assessment tools. In fact, the FRAM-LAB and PCRE estimate the risk for both fatal and non-fatal CVD events whereas the SCORE-H estimates the risk of fatal CVD events and FRAM-ATP-III estimates the risk for hard CHD. However, we believe that the agreement measure is not sensitive to this issue as it is based on the rank of the risks rather than their absolute values.

## Conclusion

Our analysis has shown poor agreement between six commonly used tools for estimating and classifying the risk of CVD in the UAE population. This demonstrates the difficulty of choosing any of these tools for clinical decision-making and public health interventions with regard to the primary prevention of CVD in the country. Applying these risk assessment tools in clinical practice could lead to a considerable variability in identifying subjects at high risk of CVD and consequently those eligible for therapeutic interventions. Owing to the high burden of CVD in the UAE, there is an urgent need to improve the accuracy of the existing CVD risk assessment tools either by recalibrating and validating them or by generating newer tools based on the longitudinal data from this region.
